# Antimicrobial Drug Resistance in Peru

**DOI:** 10.3201/eid1803.100878

**Published:** 2012-03

**Authors:** Coralith García, Gertrudis Horna, Elba Linares, Rafael Ramírez, Elena Tapia, Jorge Velásquez, Verónica Medina, José Guevara, Martha Urbina, Silvia Zevallos, Nelva Espinoza, Frine Samalvides, Jan Jacobs

**Affiliations:** Universidad Peruana Cayetano Heredia, Lima, Peru (C. García, G. Horna, F. Samalvides);; Hospital Edgardo Rebagliatti Martins, Lima (E. Linares);; Hospital Guillermo Almenara, Lima (R. Ramírez);; Hospital Nacional Cayetano Heredia, Lima (E. Tapia, F. Samalvides);; Hospital Nacional Arzobispo Loayza, Lima (J. Velásquez);; Hospital Alberto Sabogal, Lima (V. Medina);; Hospital Nacional Daniel A. Carrion, Lima (J. Guevara);; Hospital Nacional Maria Auxiliadora, Lima (M. Urbina);; Hospital Nacional Sergio Bernales, Lima (S. Zevallos);; Hospital Nacional Hipólito Unanue, Lima (N. Espinoza);; Institute of Tropical Medicine Antwerp, Antwerp, Belgium (J. Jacobs)

**Keywords:** antimicrobial esistance, methicillin-resistant Staphylococcus aureus, gram-negative bacteria, bacteria, South America, Peru, MRSA, staphylococci

**To the Editor:** In Latin American countries, rates of antimicrobial drug resistance among bacterial pathogens are high. Data on these rates in Peru are incomplete (*1*), and no institution in Peru has participated in multinational surveillance studies (*2*–*4*). To document the antimicrobial drug resistance profile of key pathogens, we organized a surveillance network of clinical laboratories from 9 hospitals (public, general, tertiary care, and quaternary care) in Lima, the capital of Peru. Over a 12-month period (April 2008–March 2009), we consecutively collected positive bacterial blood culture isolates (other than coagulase-negative staphylococci) from each of the 9 hospitals. Only the first isolate per patient was included. Patients’ age and hospital ward were recorded. Identification and susceptibility testing were performed at the Institute of Tropical Medicine Alexander von Humboldt (Lima, Peru).

*Staphylococcus aureus* was identified by conventional methods, and susceptibility testing was conducted by oxacillin salt agar screening and disk diffusion (*5*). For gram-negative bacilli, including extended-spectrum β-lactamases (ESBL), identification and susceptibility testing were performed by conventional techniques and by MicroScan NC50 panels (Dade-Behring, West Sacramento, CA, USA) (*5*). American Type Culture Collection strains were used as controls.

During the study period, we collected 1,681 unique isolates. We report the first 934 isolates tested from the more common species collected (375 *Staphylococcus aureus*, 321 *Klebsiella pneumoniae*, 125 *Escherichia coli*, and 113 *Pseudomonas aeruginosa*).

Overall, *S. aureus* was the most frequently recovered species, accounting for 22.0% of organisms. Of 375 *S. aureus* isolates tested, 244 (65.0%) were methicillin resistant (MRSA) and 131 were methicillin susceptible. MRSA frequency was highest among isolates from intensive care units (ICUs) (61 [68.5%] of 89 isolates), but it was also high among isolates from emergency wards (55 [57.3%] of 96 isolates); this difference did not reach statistical significance. Among the 244 MRSA isolates, 170 (69.6%) were also co-resistant to the combination of ciprofloxacin, gentamicin, and clindamycin; rates of co-resistance did not differ significantly among MRSA isolates from patients in the emergency ward (32/55, 58.2%) and those from patients in ICUs and hospital wards (133/184, 72.3%, p = 0.67). Among the 131 methicillin-susceptible isolates, resistance rates were as follows: ciprofloxacin (5.3%), gentamicin (10.7%), clindamycin (14.5%), and erythromycin (14.5%). All *S. aureus* isolates were susceptible to linezolid, teicoplanin, and vancomycin; clindamycin-inducible resistance was found in 10 (38.5%) of 26 isolates resistant to erythromycin and apparently susceptible to clindamycin.

*K. pneumoniae* was the second most frequently recovered organism, accounting for 19.1% of organisms collected. Among 321 *K. pneumoniae* isolates tested, 241 (75.1%) produced ESBL, 207 (64.5%) showed resistance to ciprofloxacin, and 233 (72.6%) were resistant to trimethoprim-sulfamethoxazole; proportions did not differ among age groups, wards, or hospitals. Of the 241 ESBL-producing isolates, 136 (56.4%) showed co-resistance to ciprofloxacin and gentamicin and 66 (27.4%) were also resistant to amikacin. Of the 80 non–ESBL-producing isolates, 37 (46.3%) were resistant to ciprofloxacin. All *K. pneumoniae* isolates retained susceptibility to imipenem and meropenem. A large proportion of *K. pneumoniae* infections were suspected to have been hospital acquired because most (280/321, 87.2%) were recovered from patients already hospitalized, including one third (96/321, 29.9%) of those from the neonatal ward. Although *K. pneumoniae* occasionally caused microepidemics in neonatal wards, most isolates were recovered randomly over time and from different hospitals.

Among 125 *E. coli* isolates tested, 96 (76.8%) produced ESBL, 107 (85.6%) were resistant to ciprofloxacin, and 108 (86.4%) were resistant to trimethoprim-sulfamethoxazole. The resistance rate to ciprofloxacin was higher among adults than children (90.5% vs. 60.0%, p = 0.002). Of 96 ESBL-positive isolates, 59 (61.5%) were co-resistant to gentamicin and ciprofloxacin but only 9 (9.4%) were resistant to amikacin. Among 29 non–ESBL-producing *E. coli* isolates, 19 (65.5%) were resistant to ciprofloxacin. All isolates were susceptible to imipenem and meropenem. We hypothesized that the high level of *E. coli* resistance to ciprofloxacin may be related to community overuse of fluorquinolones for common infections, such as acute diarrhea.

Among 113 *P. aeruginosa* isolates tested, 62 (54.8%) came from patients in ICUs and 73 (64.0%) were isolated from adults. Multidrug-resistance (defined as resistance to at least 3 of the following: ciprofloxacin, imipenem, amikacin, ceftazidime) was found for 67 (59.3%) of the 133 isolates, more among adults (65.7%) than among children (43.2%, p = 0.024). Overall, 34.5% were resistant to piperacilin-tazobactam.

Our main study limitation was not having complete clinical and epidemiologic information to define which isolates were acquired in the hospital and which were acquired in the community. Overall, rates of antimicrobial drug resistance among common pathogens in hospitals of Lima, Peru, were high.

## References

[1] Seas C, Hernandez K, Ramos R, Bazan E, Rodriguez I, Torres A, Oxacillin-resistant and multidrug-resistant *Staphylococcus aureus* in Lima, Peru. Infect Control Hosp Epidemiol. 2006;27:198–200. 10.1086/500650 16465640

[2] Rossi F, Baquero F, Hsueh PR, Paterson DL, Bochicchio GV, Snyder TA, Gram-negative bacilli isolated from patients with intra-abdominal infections worldwide: 2004 results from SMART (Study for Monitoring Antimicrobial Resistance Trends). J Antimicrob Chemother. 2006;58:205–10. 10.1093/jac/dkl199 16717055

[3] Diekema DJ, Pfaller MA, Schmitz FJ, Smayevsky J, Bell J, Jones RN, ; SENTRY Participants Group. Survey of infections due to *Staphylococcus* species: frequency of occurrence and antimicrobial susceptibility of isolates collected in the United States, Canada, Latin America, Europe, and the Western Pacific region for the SENTRY Antimicrobial Surveillance Program, 1997–1999. Clin Infect Dis. 2001;32:S114–32. 10.1086/320184 11320452

[4] Andrade SS, Jones RN, Gales AC, Sader HS. Increasing prevalence of antimicrobial resistance among *Pseudomonas aeruginosa* isolates in Latin American medical centres: 5 year report of the SENTRY Antimicrobial Surveillance Program (1997–2001). J Antimicrob Chemother. 2003;52:140–1. 10.1093/jac/dkg270 12775681

[5] Clinical and Laboratory Standards Institute. Performance standards for antimicrobial susceptibility testing. Document M100–S18. Wayne (PA): The Institute; 2008.

